# Nora’s lesion of the second toe

**DOI:** 10.4103/0019-5413.65150

**Published:** 2010

**Authors:** SS Suresh

**Affiliations:** Department of Orthopedics, IBRI Regional Referral Hospital, PO Box 46, IBRI 516, Sultanate of Oman

**Keywords:** Bizarre parosteal osteochondromatous proliferation, Nora’s lesion, tumor phalanx of foot

## Abstract

Bizarre parosteal osteochondromatous proliferation, otherwise known as Nora’s lesion, is surface tumor of bone with high probability of local recurrence. The author reports a case of Nora’s lesion of the proximal phalanx of the second toe, successfully managed by en bloc excision of the swelling. At four-year follow-up there was no evidence of recurrence.

## INTRODUCTION

Bizarre parosteal osteochondromatous proliferation (BPOP) is a rare tumor affecting proximal and middle phalangs of the hands and feet.^1^–^7^ There are many reports of BPOP in the long bones like humerus, femur, tibia, fibula, radius, ulna and also in the maxilla, parietal bone, and sesamoids.^1^,^2^,^4^,^8^ Clinicians and pathologists should be familiar with this rare condition as incomplete excision may lead to recurrence which has been reported to be around 50% in few reports.^1^,^6^,^9^

## CASE REPORT

**Figure 1:**
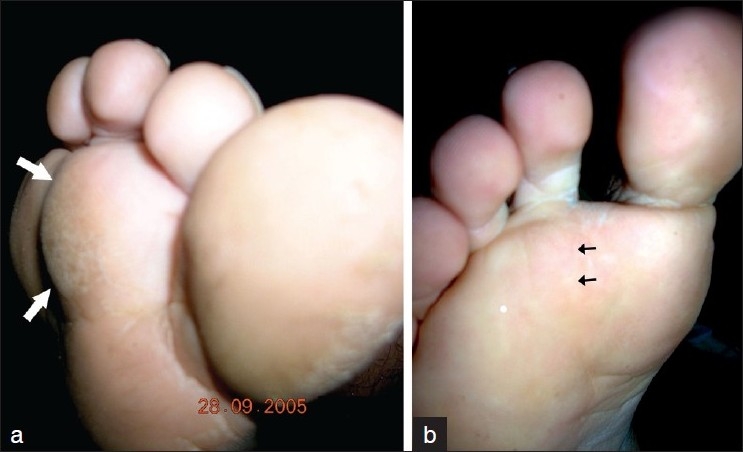
Clinical photographs showing (a) the lesion over plantar aspect of 2^nd^ toe (arrow) (b) faint scar (black arrow) at four-years follow-up

A 17-year-old boy presented in the orthopedic clinic with complaints of swelling plantar aspect of the right second toe. There was no history of trauma. The swelling was nontender and adherent to the underlying bone. The swelling was firm to hard in consistency and measured about 2.5 cm × 2.5 cm, and extended to the 2^nd^ web space [Figure 1a].

**Figure 2:**
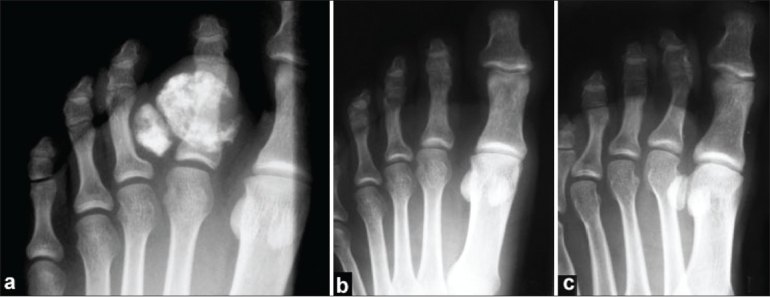
(a) Antero-posterior radiograph of the right foot showing two calcified lesions in relation to the proximal phalanx of the 2^nd^ toe. Radiograph of the right foot (b) anteroposterior view, and (c) oblique view, showing no recurrence at four years follow-up

Plain radiographs of the foot showed two radio opaque shadows over the plantar aspect of the right foot, more towards the 2^nd^ inter digital cleft, but a small part in the 1^st^ inter digital cleft. The biggest of the lesion was almost round and found encircling the entire diaphysis of the proximal phalanx of the right 2^nd^ toe. This had fluffy calcification. There was another sclerotic mass in the 2^nd^ inter digital space, almost ovoid in shape [Figure 2a].

Under general anesthesia and tourniquet control the patient underwent surgical excision of the lesion, through a plantar based incision. There was a multi-lobulated swelling, which was bluish white in appearance, and measuring 4 × 2.5 × 2 cm. The entire tumor was shaved off from the shaft of the proximal phalanx. Cut section of the mass showed grayish white areas at the periphery and granular bony tissue below it, with areas of chalky white appearance in between.

**Figure 3:**
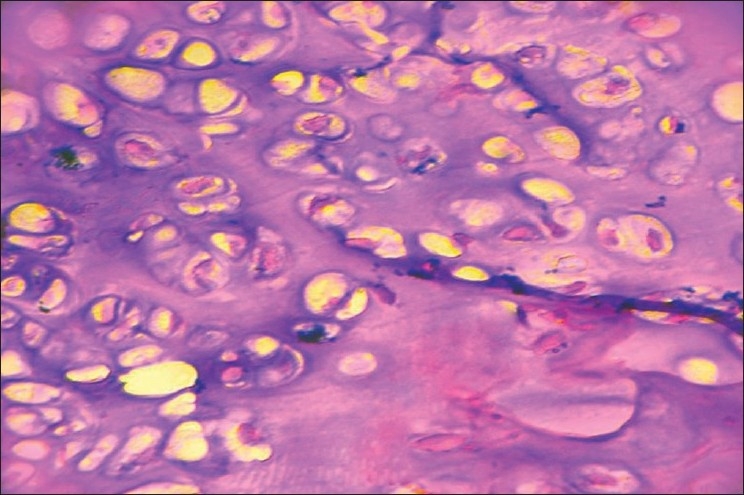
Histopathology picture showing atypical chondrocytes and binucleated cell

Histopathology showed a mixture of cartilage, fibrous tissue and bone. The chondrocytes were ‘bizarre’ and irregularly arranged, with occasional bi-nucleated cells [Figure 3]. A diagnosis of “bizarre parosteal osteochondromatous proliferation” was made and the probability of recurrence was explained to the patient. He was followed up regularly and at four years follow-up, the patient was symptom-free and without local recurrence [Figure 1b, 2b, c].

## DISCUSSION

First described by Nora *et al*. [hence known as Nora’s lesion], BPOP is a benign surface tumor from the small tubular bones of the hands and feet.^1^–^6^ This presents as a slow growing mass, progressing over a period of a few months to two years. There may be a history of trauma in around 20% of cases, a traumatic episode triggering a localized proliferation of periosteum. Ossification in BPOP may resemble callus tissue at the bone cartilage interface and many authors consider trauma as a cause.^10^,^11^

Mostly a tumor of the middle and proximal phalanges of the hand,^2^,^3^,^6^,^9^,^11^ the lesion has been reported from long bones and also in the skull.^1^,^8^ More than 160 cases have been reported in the literature so far.^2^ Age of onset ranges from 8-74 years, 2,4,6 with most of the cases occurring between 20-35 years, with equal sex predilection.^10^

BPOP arise directly from the cortical surface of the bones, as if they are “stuck” on the periosteum.^9^ The underlying bone is structurally normal without cortical flaring as in osteochondroma.^8^ In radiographs they appear as well defined mass, though in some cases they may exhibit speculated or irregular surface as in our case causing diagnostic dilemma.^10^ There is no continuity for the lesion with the medullary cavity of the host bone. Intra lesional calcifications may be seen in some of the lesions. Absence of medullary continuity is better visualized in a CT scan.^1^,^2^,^9^

These are single or multiloculated lesions, macroscopically, with a diameter ranging from 05-40 mm.^6^ The whole lesion usually appears bluish giving the “blue bone” appearance.^1^,^2^,^9^

There is bizarre intermingling of cartilage, bone and fibrous tissue. The cartilage is composed of hyper cellular chondrocytes, some of which are binucleated. There are no atypical fibrous spindle cells, thus differentiating BPOP from parosteal osteosarcoma.

A differential diagnosis of parosteal osteosarcoma, osteochondroma, myositis ossificans, and benign florid periostitis is considered. Initial diagnosis in many reports is osteochondroma because of the parosteal location, cartilage cap and histological presence of chondrocytes.^9^,^10^ Osteochondroma grow towards the physis and there is medullary continuity with the lesion.^1^,^2^ Also, osteochondroma is rare in the small bones of the hand.

It is a highly aggressive tumor with local recurrence reported as high as 50%.^1^,^3^,^6^,^8^,^9^ Recurrence occurs between two months to two years. En-bloc negative margin excision, with complete removal of the pseudo-capsule, resection of any periosteal tissue underneath the tumor and decortication of the underlying cortex is likely to prevent recurrence.^2^ Even in cases of recurrence a repeat excision is advocated, rather than aggressive surgery.^10^ Our case was followed up for four years and there was no evidence of recurrence.
